# Characterization of melanic and non-melanic forms in domestic and peridomestic populations of *Triatoma infestans* (Hemiptera: Reduviidae)

**DOI:** 10.1186/s13071-020-3912-y

**Published:** 2020-02-03

**Authors:** Julieta Nattero, Ana Laura Carbajal de la Fuente, Romina Valeria Piccinali, Miriam Cardozo, Claudia Susana Rodríguez, Liliana Beatriz Crocco

**Affiliations:** 1 Departamento de Ecología, Genética y Evolución/Laboratorio de Eco-Epidemiología, Facultad de Ciencias Exactas y Naturales, Universidad de Buenos Aires, Buenos Aires, Argentina; 2 Instituto de Ecología, Genética y Evolución (CONICET-IEGEBA), CONICET-Universidad de Buenos Aires, Buenos Aires, Argentina; 3 Cátedra de Introducción a la Biología, Facultad de Ciencias Exactas, Físicas y Naturales, Universidad Nacional de Córdoba. Instituto de Investigaciones Biológicas y Tecnológicas (IIBYT-CONICET), Avda. Vélez Sarsfield 299, piso 5, X5000JJC Córdoba, Argentina

**Keywords:** Colorimetric analysis, Developmental instability, Flight initiation, Melanic/non-melanic, Morphological differences, Selective advantage, *Triatoma infestans*

## Abstract

**Background:**

Melanic (dark) morphs have been barely reported in peridomestic and sylvatic conditions for *Triatoma infestans*, the most important vector of Chagas disease in the Southern Cone of South America. Adults with dark and small yellow markings on the connexivum were collected after manual searches conducted by technical personnel in 62 domiciliary units in Cruz del Eje, Córdoba Province, Argentina. The last community-wide insecticide spraying campaign before the study had been conducted three years earlier. We investigated if there was a measurable color morph variation (melanic and non-melanic) in wings and connexivum; we determined infestation, distribution of melanic and non-melanic forms, and correspondence of colorimetric variation with variations in morphology (wing size and shape and body length), development (wing fluctuating asymmetry), physiology (nutritional status) or behaviour (flight initiation).

**Results:**

Forty-nine females, 54 males and 217 nymphs were collected in 24 domiciliary units. House infestation and colonization were 53% and 47%, respectively. Most of the *T. infestans* individuals (83.2%) were collected in chicken coops; intradomicile infestation was recorded in only one case. The chromatic cluster analysis showed two well-defined groups: melanic and non-melanic. The melanic group included 17 (35%) females and 25 (46%) males. Peridomestic infestation was lower for melanic than for non-melanic adults. Melanic morphs were collected in houses from several localities. Sexual dimorphisms were confirmed by morphometric measurements. Body length was large in melanic adults (*P* < 0.01 only for males). Differences between groups were significant for wing size and shape, but not for weight or weight/body length ratio. Melanic females and males showed significantly higher fluctuating asymmetry (FA) indices than their non-melanic counterparts.

**Conclusions:**

This is the second report of melanic forms of *T. infestans* in domestic and peridomestic habitats in the Dry Chaco region of Argentina. Although non-melanic adults exhibited a higher infestation rate, melanic adults were widespread in the area and were collected in the infested domicile and in most types of peridomestic annexes. Differences in morphometric variables between groups might be due to different ecological adaptations. The higher FA levels observed in melanic individuals suggest a higher developmental instability and a selective advantage of non-melanic individuals in domestic and peridomestic habitats. 
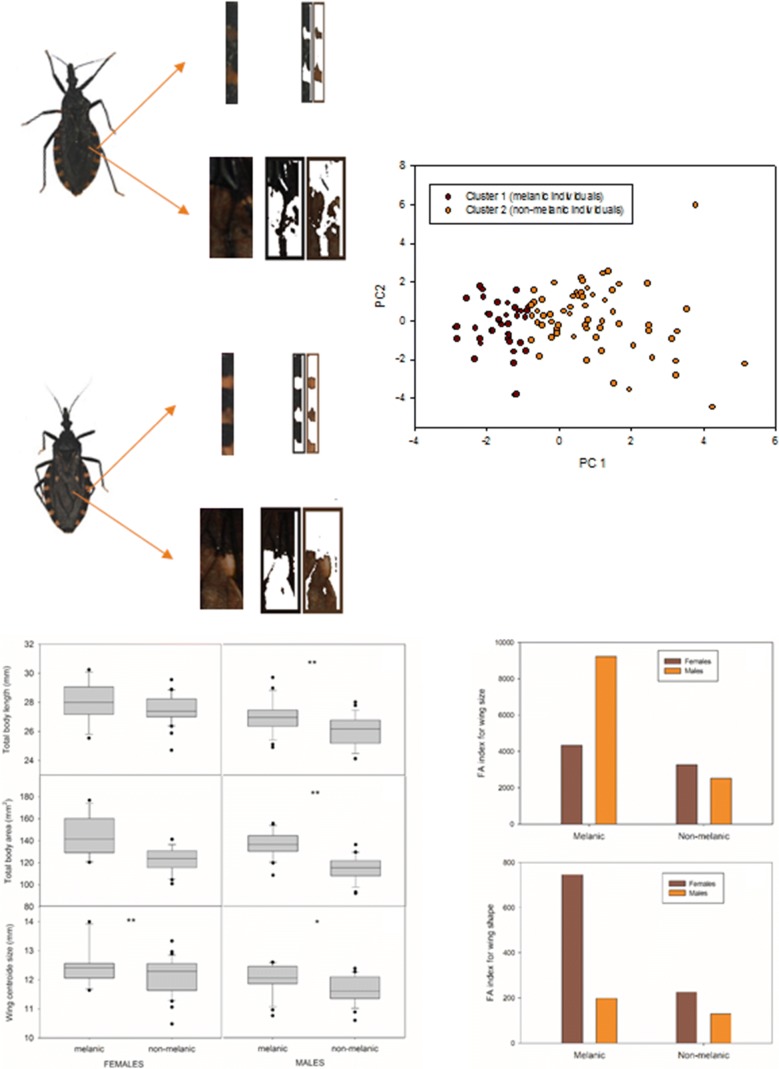

## Background

Natural populations often exhibit great phenotypic variation. Differences in visible traits among organisms, such as body color, are the most conspicuous evidence of morphological variability in nature. One of the simplest and most common examples of such conspicuous variation is melanism, the occurrence of variants that are mostly or completely dark in pigmentation. This type of variation may involve discrete melanic and non-melanic phenotypes or continuously varying pigmentation [1]. Melanism is very common in insects, with melanic or dark morphs exhibiting an unusually high concentration of cuticular melanin [2]. Melanin pigments and their precursors are important structural and protective components of the cuticle [3]. Diverse selective pressures, including visual and non-visual cues, might be associated with melanism, (reviewed in [1]). Theory posits that individuals with dark color patterns would be at an advantage under low ambient temperature and high UV radiation conditions and should be less tolerant to desiccation [4–7]. Melanism also has correlated and/or pleiotropic effects, including morphological, developmental, physiological, behavioural and/or neurological traits (reviewed in [1]).

*Triatoma infestans* (Hemiptera: Reduviidae: Triatominae) is the most important vector of *Trypanosoma cruzi*, the etiological agent of Chagas disease, in the Southern Cone of South America [8]. Insecticide spraying reduced the geographical range and abundance of *T. infestans* but did not interrupt vector transmission of *T. cruzi* in the Gran Chaco [9], a 1.3 million-km^2^ ecoregion extending across Paraguay, Bolivia and Argentina, including the northwestern extreme of Córdoba Province, Argentina [10]. The northwest of Córdoba Province has been historically an endemic Chagas disease region. The latest epidemiological reports from this disease in Argentina indicate Córdoba as one of the provinces with the highest risk of vector and congenital transmission of *T. cruzi* [11].

Although color pattern quantification has been performed in other insect species (e.g. the local mimicry polymorphism of *Heliconius* butterflies, [12]), in Triatominae species, color variation has not yet been quantified. Melanic (dark) morphs of *T. infestans* were reported for the first time in sylvatic foci of the Bolivian Chaco in an isolated dry forest very far from human settlements [13, 14]. Morphologically, these specimens were very similar to domestic *T. infestans* collected from the Bolivian Chaco, except for their overall darker coloration with small yellow markings on the connexivum [13]. Melanic forms were then reported near chicken coops in the humid forest of northeastern Argentina (an area not included in the Gran Chaco) [15], in fallen trees among shrubs close to an indigenous community in the Paraguayan Chaco [16] and in hollow tree trunks harboring parrot nests in the Argentine Dry Chaco [17]. Recently, domestic colonies of the dark morph were reported in the Chaco Province from Argentina [18]. Several studies using different methods confirmed that individuals of the dark morph were chromatic variants of *T. infestans* differing in color, head and wing morphometry, antennal sensilla patterns, chromosome C-banding, nuclear rDNA sequences, genome size, and mitochondrial cytochrome B and CO1 genes [13, 17, 19–24].

Several *T. infestans* adults collected in domestic and peridomestic habitats in Cruz del Eje, Córdoba Province, Argentina, in 2012 displayed an evident visual variation in color, with some individuals being darker than others and having small yellow markings on the connexivum. To our knowledge, there are only one record of melanic morphs in domestic and peridomestic populations of *T. infestans* from the Gran Chaco of Argentina [18]. One of the most important current challenges of interrupting Chagas disease transmission is to know the origin of reinfestant insects after the application of vector control actions. Recent studies have shown that *T. infestans* populations that remain after spraying campaigns are highly connected to sylvatic individuals that could be involved in restoring the reinfestation process [25]. These data show the importance of sylvatic *T. infestans* populations in recolonization of treated areas. Considering that melanism could be the result of an adaptation to certain environmental conditions, melanic individuals might be more adapted than non-melanic individuals to live in human-made structures, such as peridomiciles, which are less isolated from the surrounding environment and more exposed to harsh weather.

The main goal of this study was to analyze this particular chromatic polymorphism of *T. infestans*, its relationship with infestation parameters and its correlation with other variables. Particularly, we aimed to (i) investigate if there is measurable variation in color morphs (melanic and non-melanic) in adults collected in domestic and peridomestic habitats; (ii) determine infestation, frequency and distribution of melanic and non-melanic forms; and (iii) determine if colorimetric variation corresponds to other variations in morphology (wing geometric morphometry, total body length and total body area), development (wing developmental instability), physiology (nutritional status) and behaviour (flight initiation). Wing shape analyses performed to assess population structuring in *T. infestans* showed patterns compatible with independent geographical origins [25, 26] or the presence of different groups or variants [21, 27]. A different nutritional status between melanic and non-melanic forms suggests dissimilar feeding capacity and ability [28, 29]. Variations in the total weight/total body length (W/L) ratio were related to different flight initiation probabilities [30–33]. Differences in wing developmental instability may indicate differences in ecological adaptation to the environmental conditions of the habitat where the insects develop [34, 35]. We expected to find differences in morphometric traits between melanic and non-melanic groups as proxies of differences in their ecological adaptations.

## Methods

### Study sites

**Fig. 1:**
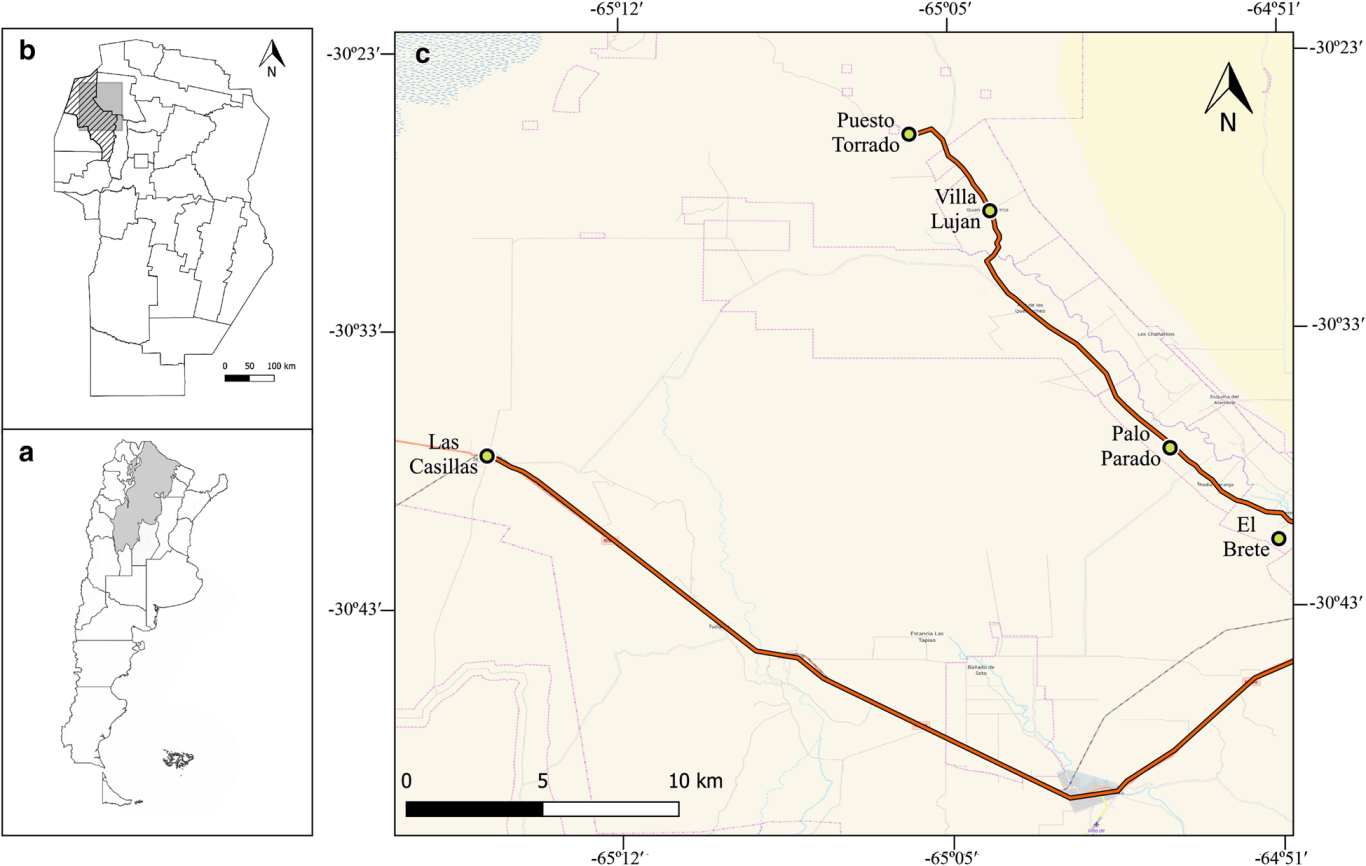
Maps of the study area. **a** Córdoba Province (light grey) and Chaco region (dark grey) in Argentina. **b** Cruz del Eje department, Córdoba Province, Argentina. **c** Individual collection localities

All the insects included in this study were collected in a rural area (approximately 60 × 50 km) of Cruz del Eje department, Córdoba Province, northwestern Argentina, in December 2012 (Fig. 1). This area is part of the Argentine Dry Chaco, characterized by a subtropical dry climate with a summer season from October to March. The last community-wide insecticide spraying campaign before this study had been carried out by vector control personnel three years earlier. The present cross-sectional study was conducted in domiciliary units, which consisted of a domicile with human resting places and the associated peridomicile, usually chicken coops, goat or pig corrals and, less frequently, rabbit hutches or storerooms. Personnel of the National Vector Control Programme inspected 62 houses for the presence of *T. infestans*. After inspection, technicians sprayed all the infested houses with cypermethrin.

### Insects

Triatomines were searched for inside domiciles and peridomestic structures using the hour-person technique [36]. For this purpose, one person searched for the presence of triatomines at each site within each house during 20 min. All collected insects (103 adults and 217 nymphs) were taken to the laboratory under refrigerated conditions and species, sex and stage were identified [37]. All *T. infestans* adults were evaluated for the presence of *T. cruzi* in feces using a conventional optical microscope; none of them were positive for the presence of the parasite.

Weight (W) of adults was recorded on an electronic scale (precision ± 0.001 g; Mettler, Denver, USA) the day after collection. Dorsal view digital photographs of all adults were taken under a white background with a reference scale under the same illumination conditions and camera position. After insect dissection, right and left wings of each individual were mounted on graph paper. Digital photographs of wings were taken with a Moticam 2 (CMOS, Richmond, Canada) camera connected to a stereomicroscope (Stemi 2000-C; Zeiss, Oberkochen, Germany) under 6 × magnification.

### Infestation by *T. infestans*

A house was recorded as colonized when at least one *T. infestans* nymph was found in the evaluated site (intradomicile or peridomicile structure). We considered infestation or colonization in the intradomicile (IDI, IDC) or in the peridomicile (PDI, PDC) when *T. infestans* adults and/or nymphs were recorded inside the domicile or in the peridomicile structures, respectively. House infestation was calculated as the percentage of houses infested with adults only, with adults and nymphs, or with nymphs only of the total evaluated houses. House colonization was calculated as the percentage of colonized houses of the total of evaluated houses. Infestation was also estimated for melanic and non-melanic forms.

### Colorimetric analysis

**Fig. 2:**
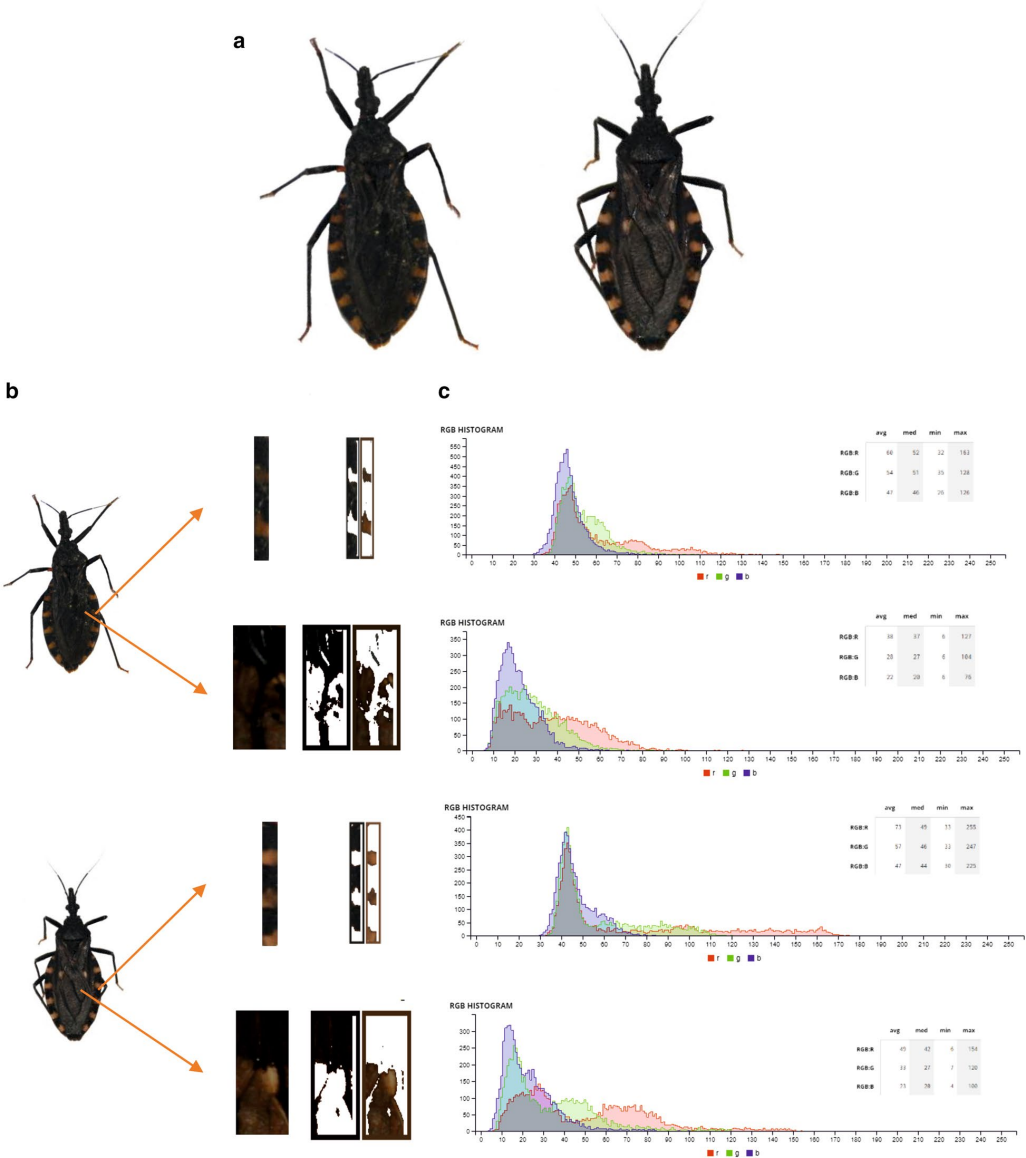
Example of colorimetric analysis of wings and connexivum of *T. infestans* adults collected in Cruz del Eje, Córdoba Province, Argentina. **a** Example of a collected melanic (left) and non-melanic (right) females. **b** Portions of right wing and connexivum subjected to colorimetric analysis. **c** RGB histogram derived from portions of wing and connexivum for melanic and non-melanic forms

Color was quantified from image data of wings and connexivum (Fig. 2a). Consistent comparisons of color from images requires homologous alignment of the portion of tissue to be analyzed and color-based segmentation of the images [38]. We performed homologous alignment of a portion of right wing and connexivum by extracting 8.5 × 20 mm rectangles from the wing and 1.5 × 18 mm rectangles from the right side of the connexivum (Fig. 2b). We chose the wing to quantify color because we observed the presence of a yellow spot on the upper third of the wings only in the clearer individuals. The colorimetric analysis was performed using the software Image Color Summarizer 0.76 (http://mkweb.bcgsc.ca/color-summarizer/). We obtained the average value of each red green blue (RGB) component, as three separate variables, for the portions of wing and connexivum of each individual (six variables in total). A K-means clustering analysis was used to define the number of colorimetric groups present in the sampled *T. infestans* populations [39]. Euclidean distance measure was selected for the cluster analysis. The K-means clustering algorithm was run *a priori* considering up to 10 clusters. The optimal number of clusters for the data set was determined using the elbow and average silhouette methods [40]. The elbow method looks at the percentage of explained variance, the sum of squared errors, as a function of the number of clusters. The chosen number of clusters is the one that does not improve with the addition of a new cluster. The average silhouette method considers that the optimal number of clusters is the one that maximizes the mean silhouette value, a measure of how similar an object is to its own cluster compared to other clusters [40].

### Body data collection

Images of a dorsal view of each individual were processed using the UTHSCSA ImageTool for Windows ver. 3.00. Total body length (L) was measured from the base of the head to the base of the body. Body area (mm^2^) was calculated excluding the head, pronotum and scutellum. The relationship between W and L was also estimated as the W/L ratio.

### Wing data collection

We used a landmark-based geometric morphometry approach to collect 10 type-I landmarks positioned at wing vein intersection, as described elsewhere [27]. Landmarks were collected using TPSdig 2.31 [41]. Comparison of wing size and shape between melanic and non-melanic groups for each sex involved the right wing.

To assess fluctuating asymmetry (FA), left wings were first reflected to their mirror images. Separate landmark configurations were digitized twice in both wings of each individual to estimate measurement error (ME) [42]. Asymmetry can be partitioned in directional and non-directional asymmetry. Directional asymmetry (DA) occurs whenever there is, on average, a greater development of a character on one side of the plane of symmetry than on the other. DA is presumably unrelated to developmental stability [42]. Non-directional asymmetry measures FA.

### Statistical analysis

K-means clustering analysis was done using the software InfoStat [43]. The elbow and silhouette methods used to determine the optimal number of clusters were performed with factoextra R package (https://cran.r-project.org/web/packages/factoextra/index.htmll). After assigning individuals to melanic and non-melanic groups *via* K-means clustering, comparisons between females and males within each group were significant in most of the performed analyses; therefore, males and females were treated separately in the comparisons between groups.

After checking that assumptions for parametric analyses were met, W, L, W/L ratio, and body area were compared between melanic and non-melanic groups for each sex using a t-test. Wing size was measured using the centroid size (CS) variable. This measure is a single variable of size that integrates different axes of growth, and is measured as the square root of the sum of the squared distances between the centre of the configuration of landmarks and each individual landmark [44]. Wing size (CS) was compared between groups for each sex through a one-way analysis of variance (ANOVA). These analyses were done with the software InfoStat [43].

Wing shape was compared using a Procrustes approach, by computing differences of landmark coordinates after a full Procrustes fit superimposition of both sexes and groups. Comparisons were performed using discriminant function analysis (DFA), first between sexes and then between melanic and non-melanic groups for each sex. Mahalanobis distances between pairs of species were calculated and their significance was evaluated using a non-parametric test based on permutations (1000 runs). The percentage of phenotypic similarity between pairs of species was calculated using the cross-check test of discriminant analysis. The relationship between CS and shape within each group (allometry) was estimated using a multivariate regression between the Procrustes coordinates (dependent variables) and the CS (independent variable). Morphometric analysis was done using MorphoJ 1.05f [45].

To assess FA, configurations for melanic and non-melanic females and males were superimposed using the least-squares Procrustes method [46]. A two-way mixed ANOVA with side and individual as fixed and random factors, respectively was performed for each group within each sex to assess the occurrence of DA and FA [47]. A two-way mixed ANOVA for wing size and a Procrustes ANOVA for wing shape were performed in each group to estimate the occurrence of FA in size and shape, respectively [47]. FA index for size and shape was estimated as the mean square (MS) of the interaction between side and individual of the Procrustes ANOVA. These indices were corrected for measurement error (MS/ME × 10^3^).

To analyze allometry a multivariate regression was computed between the Procrustes coordinates (dependent variables) and CS (independent variable) for melanic and non-melanic individuals within each sex. MorphoJ 1.05f [45] was used for FA analysis.

## Results

### Colorimetric analysis of melanic and non-melanic forms

**Fig. 3:**
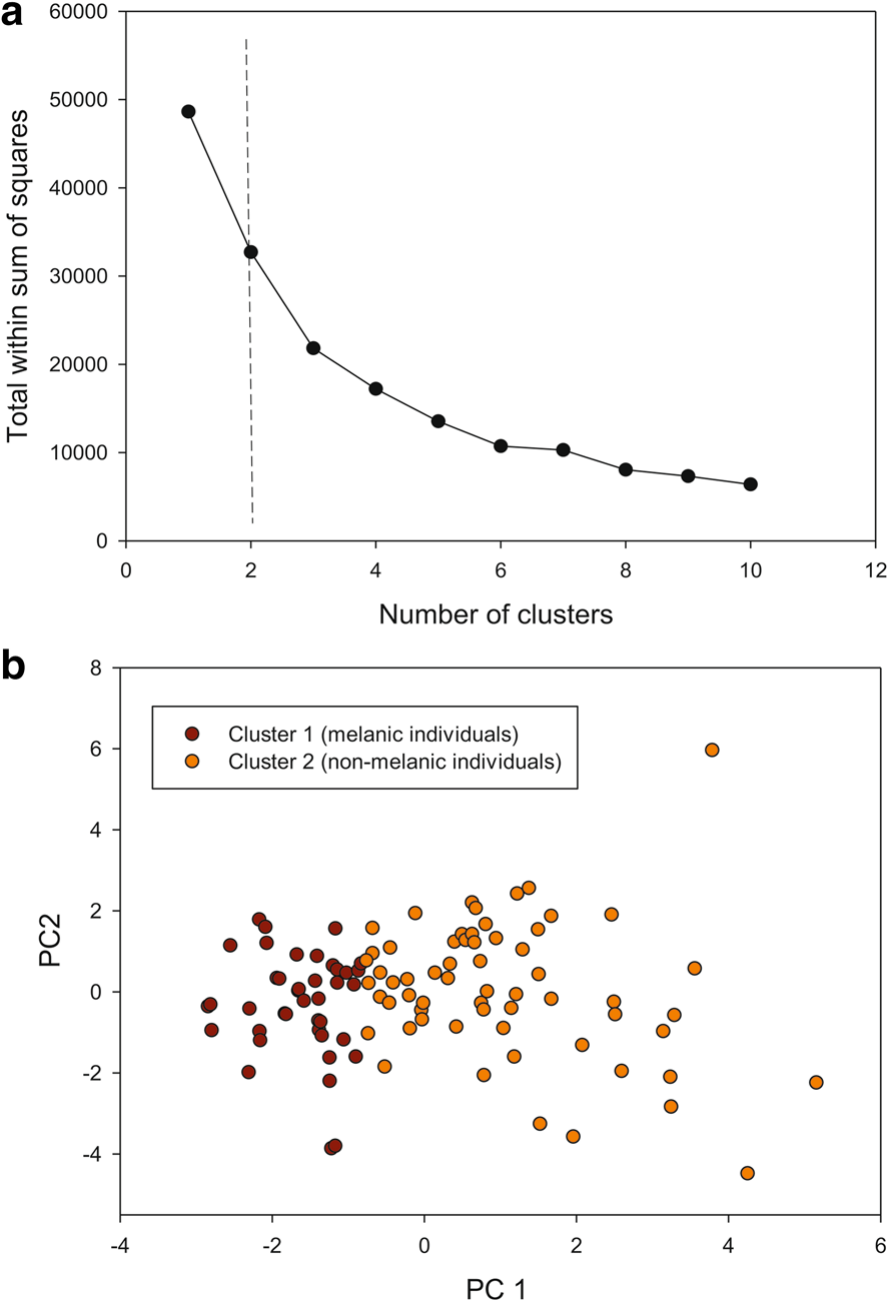
K-means clustering analysis. **a** Plot of the curve of the total within-cluster sum of square for 10 K-values (elbow method). The optimal number of clusters suggested by this method (two) is indicated with a dashed line. **b** Biplot derived from the first two axes of a principal components analysis showing the spatial distribution of the two clusters derived from a K-means clustering analysis (K = 2)

An example of the colorimetric analysis procedure is presented in Fig. 2c. The six variables derived from the RGB threshold analysis were used in the K-means clustering analysis. The number of optimal clusters, evaluated with the elbow and silhouette method, was two and three groups, respectively (Fig. 3a, Additional file 1: Figure S1a). A PCA showing the distribution of the individuals in two clusters suggested relatively well-defined melanic and non-melanic groups, with the non-melanic group being more variable than the melanic one (Fig. 3b). The first PCA axis (explaining 49.54% of the total variance) was mainly associated with wing RGB channel colors and the second axis (explaining 45.77% of the total variance), was mainly associated with connexivum RGB colors (Table 1). When three clusters were graphed in a biplot for the first two PCA axes, the melanic group remained the same, whereas the non-melanic group was split into two groups (Fig. 3b, Additional file 1: Figure S1b). For this reason, we chose the criterion of two clusters (melanic and non-melanic groups) for the subsequent analyses.

**Table 1:** Eigenvectors of a principal components analysis derived from a colorimetric analysis of connexivum and wing for *T. infestans* adults collected in Cruz del Eje, Cordoba Province, Argentina

Structure	Channel color	PC1 (49.54%)	PC2 (45.77%)
Wing	Red	0.441	− 0.363
	Green	0.457	− 0.371
	Blue	0.447	− 0.357
Connexivum	Red	0.365	0.442
	Green	0.379	0.452
	Blue	0.346	0.452

*Abbreviations*: PC1, principal component 1; PC2, principal component 2

### House infestation and distribution, and frequency of melanic and non-melanic forms

All the studied ecotopes harbored relatively small populations, with low abundance of adults (between 1 and 18) and nymphs (between 1 and 36) of *T. infestans* (Table 2). House colonization rate was 46.77% (29/62), and house infestation rate was 53% (33/62). Only *T. infestans* adults occurred in 5 of the 33 infested houses. An established colony was collected in the intradomicile in a single domiciliary unit (IDI = 4.17%, IDC = 4.17%) (Table 2). For peridomestic habitats, PDI was 37.10 % and PDC 43.54 %. One house was infested in more than one peridomestic structure (Table 2). Most (83.2%) *T. infestans* individuals (161 nymphs and 81 adults) were collected in chicken coops, whereas the remaining ones (17.8%, 37 nymphs and 16 adults) were collected in goat corrals, pig corrals and storerooms. A rabbit hutch harbored only one male of *T. infestans* (Table 2).

**Table 2:** Number of *Triatoma infestans* nymphs, adults, and melanic and non-melanic adults collected in houses of different localities from Cruz del Eje, Córdoba Province, Argentina

No. of identified houses	Locality	Ecotope	Total no. of collected nymphs	Total no. of collected adults	No. of melanic females	No. of melanic males	No. of non-melanic females	No. of non-melanic males
19	El Brete	Chicken coop	20	5	3	0	1	1
51	La Casilla	Chicken coop	36	4	0	0	1	3
52	La Casilla	Goat corral	0	2	0	0	1	1
8	Palo Parado	Rabbit hutch	0	1	0	0	0	1
10	Palo Parado	Chicken coop	22	2	2	0	0	0
10	Palo Parado	Storeroom	0	1	0	0	1	0
17	Palo Parado	Goat corral	7	2	0	0	2	0
802	Palo Parado	Chicken coop	3	10	0	0	5	5
803	Palo Parado	Chicken coop	4	3	0	2	0	1
805	Palo Parado	Chicken coop	1	5	2	0	1	2
806	Palo Parado	Chicken coop	3	3	0	0	1	2
807	Palo Parado	Chicken coop	7	6	0	0	1	5
808	Palo Parado	Chicken coop	34	1	0	1	0	0
809	Palo Parado	Chicken coop	0	18	1	12	3	2
810	Palo Parado	Chicken coop	8	2	0	0	2	0
812	Palo Parado	Chicken coop	10	6	4	0	2	0
813	Palo Parado	Chicken coop	3	6	0	0	3	3
814	Palo Parado	Chicken coop	8	3	0	0	3	0
817	Palo Parado	Pig corral	7	4	0	0	2	2
818	Palo Parado	Chicken coop	1	2	0	0	1	1
822	Palo Parado	Chicken coop	0	1	1	0	0	0
11	Puesto Torrado	Storeroom	13	1	1	0	0	0
5	Villa Lujan	Pig corral	10	6	2	3	1	0
7	Villa Lujan	Chicken coop	2	4	0	3	1	0
801	Villa Lujan	Domicile	18	5	1	4	0	0
	Total		217	103	17	25	32	29

*Notes*: The total number of infested houses was 33; this table only includes the 24 houses that presented adult infestation. The number of melanic and non-melanic adults per house was determined *via* a K-means clustering approach

Melanic individuals were present in 13 (54%) of the 24 infested houses. Of the 103 collected adults, 17 (35%) females and 25 (46%) males were assigned to the melanic morph. Houses with presence of melanic individuals were distributed in all sampled localities, except in the only sampled house from Puesto Torrado (Table 2). Melanic and non-melanic adults were collected together in the same habitat in 7 of the 13 houses with melanic individuals (Table 2). In four of the five houses that exhibited only melanic individuals, nymphs were also collected, indicating ongoing colonized sites (Table 2). In 12 of the 24 houses (50%) only non-melanic individuals were collected. Of these 12 houses, nymphs were not collected in only three (25%) (Table 2). In the only house that exhibited domiciliary infestation, all collected adults were melanic (Table 2). Adult peridomestic infestation (APDI) was lower for melanic than for non-melanic adults (APDI_melanic_ = 19.35, APDI_non-melanic_ = 32.26). Considering only infested houses, 45.83% exhibited melanic and 83.33%, non-melanic adults.

### Linear morphometric analysis and relationship with nutritional status

**Fig. 4:**
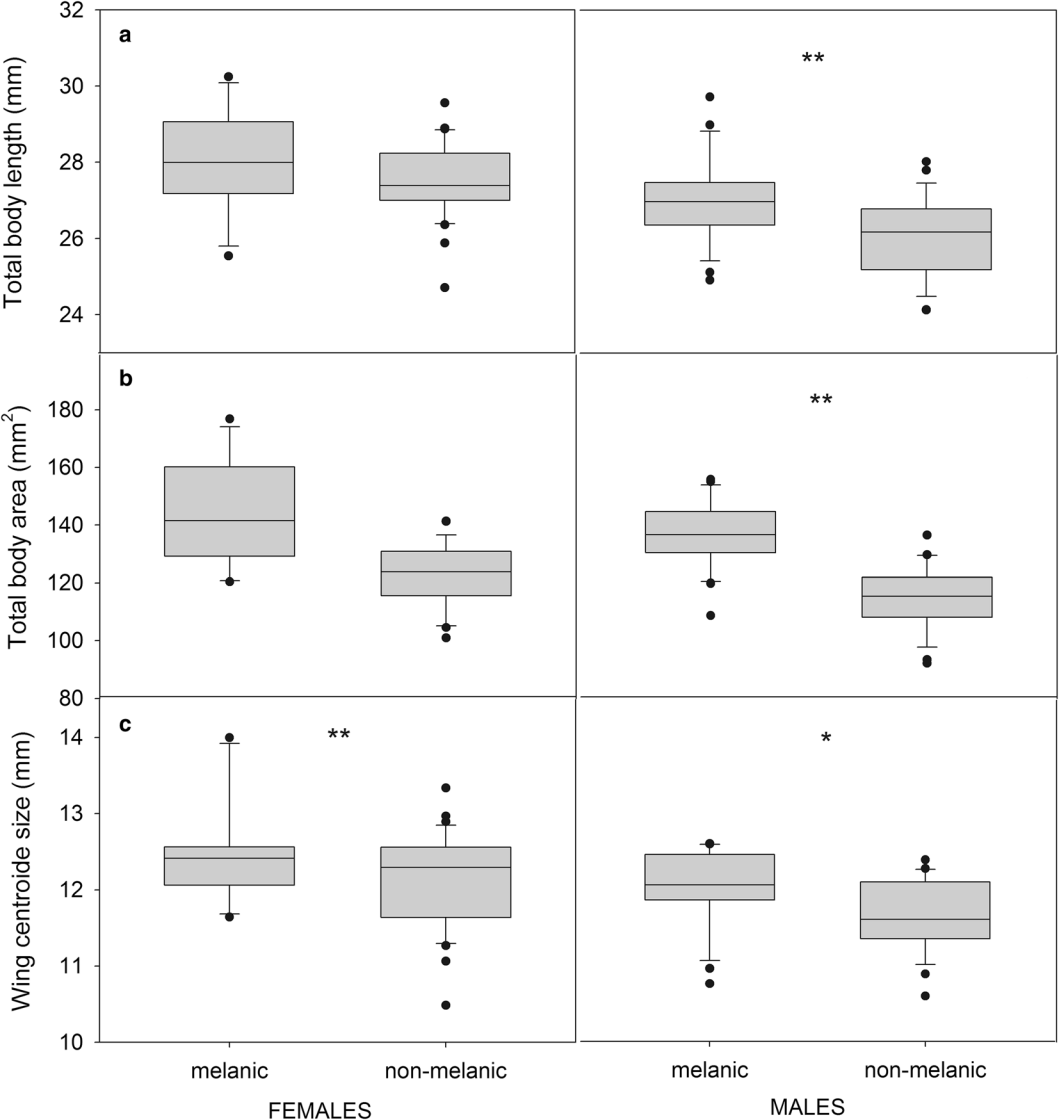
Sex-specific variations of *T. infestans* for total body length (**a**), total body area (**b**) and wing size (CS) (**c**) between melanic and non-melanic forms. Box plots show the median (solid line), first and third quartiles. Whiskers indicate the 90th and 10th percentiles. Dots outside whiskers are potential outliers. *Key:* Left box plots: females. Right box plot: males. Asterisks within graphs indicate significant differences in the ANOVA tests between melanic and non-melanic groups. *P < 0.05, **P < 0.01

Sexual dimorphism was confirmed for L, W and W/L (one-way ANOVA between sexes, *P* < 0.001 in all cases). The one-way ANOVAs for L and total body area did not show significant differences between melanic and non-melanic females (*t*_*(*45)_ = 1.38, *P* = 0.1736; *t*_(45)_ = 1.18, *P* = 0.1619 for body length and body area, respectively), although melanic females tended to have greater body length and area (Fig. 4a, b). For males, L and body area showed significant differences between groups (*t*_(48)_ = 3.27, *P* = 0.0020; *t*_(48)_ = 2.72, *P* = 0.0091, respectively). Melanic males had greater body area than non-melanic ones (Fig 4a, b). For the entire adult sample, W/L ratio varied between 7.10–17.41 mg/mm. For females, W/L varied between 9.09–14.74 mg/mm for melanic individuals and between 7.12–17.41 mg/mm for non-melanic individuals, whereas for males, W/L varied between 7.11–13.33 for melanic individuals and between 7.10–12.35 mg/mm for non-melanic individuals. W and W/L did not show significant differences between colorimetric groups, either for females or for males (*P* > 0.05 in all cases). For the houses where both colorimetric groups coexisted and each group was represented by more than one adult (i.e. houses ID No. 19, 805, 809 and 812), comparisons between W/L were made. No significant differences were found in any of the four houses (*P* > 0.05 in all cases).

### Geometric morphometric analysis

Comparisons of wing size (CS) between groups showed significant differences both for females and males (Females: *t*_(46)_ = 2.01, *P* = 0.050; males: *t*_(50)_ = 2.97, *P* = 0.005), with melanic females and males exhibiting larger wings than their non-melanic counterparts (Fig. 4c).

The first axis of the DFA for wing shape variation between melanic and non-melanic females explained the total variation (100%). Mahalanobis distances between groups were highly significant (Mahalanobis distance: 1.953, *P* = 0.0008). Non-melanic females showed the lowest misclassification error (90.91% and 84.21% of correct classification from cross-validation for non-melanic and melanic females, respectively). For males, the first axis of the DFA also explained the total variation. Mahalanobis distances were significant between groups (Mahalanobis distance: 1.496, *P* = 0.0054). Non-melanic males showed the lowest misclassification error (80.77% and 71.43% of correct classification from cross-validation for non-melanic and melanic males, respectively).

Allometric analysis performed through multivariate regressions between the Procrustes coordinates and the CS for melanic and non-melanic individuals within each sex were non-significant (*P* > 0.05 in all cases).

### Fluctuating asymmetry analysis

**Fig. 5:**
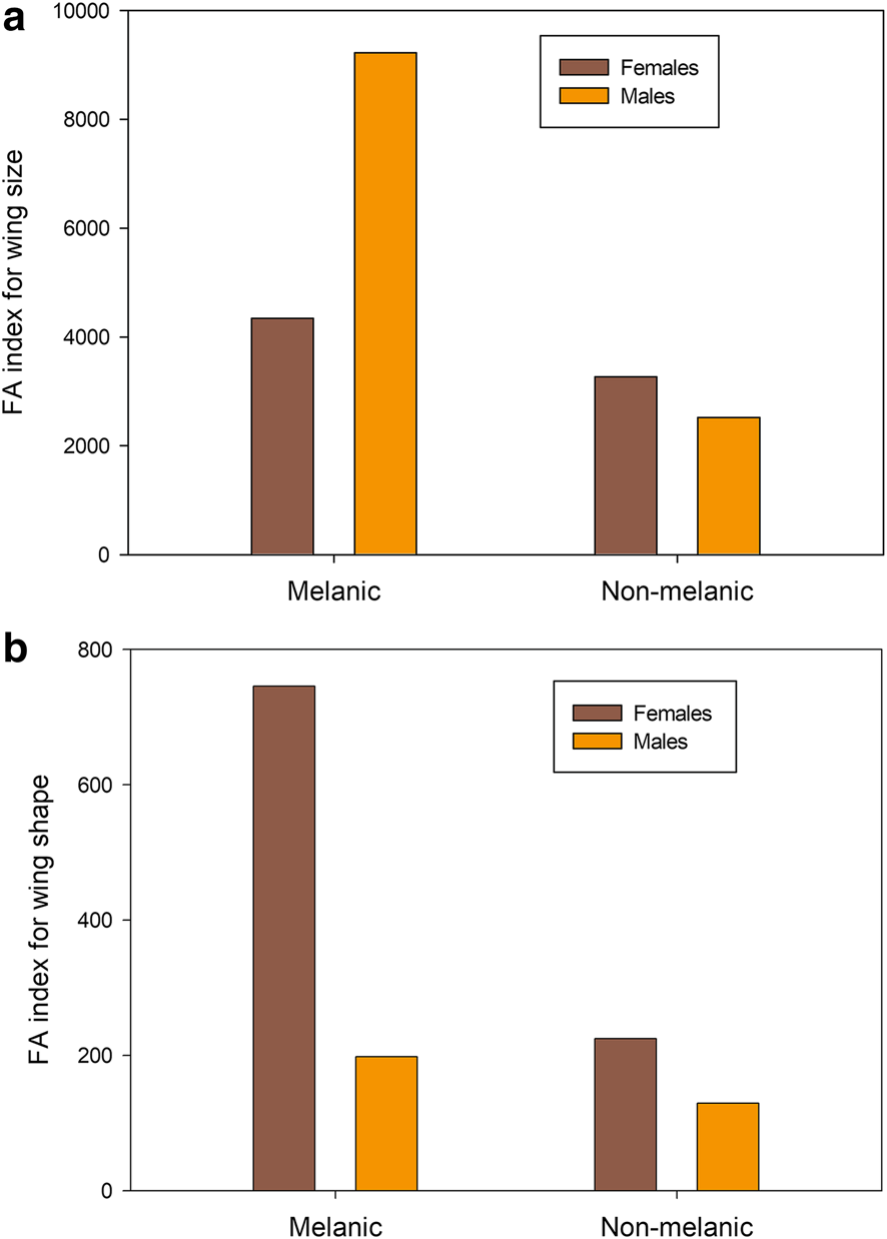
Fluctuating asymmetry index for wing size and wing shape for melanic and non-melanic females and males of *T. infestans* collected in Cruz del Eje, Córdoba Province, Argentina. FA indices represent the mean square (MS) of the side-individual interaction effect corrected for measurement error (MS/ME), as shown in the ANOVA output (Table 3). **a** Wing size FA index. **b** Wing shape FA index

Significant evidence of FA in wing size was revealed by the two-way mixed ANOVAs for melanic and non-melanic females and males (Table 3). Melanic females and males exhibited significantly higher wing size FA indices than their non-melanic counterparts (Fig. 5a). Procrustes ANOVA for wing shape FA also showed significant FA occurrence for both melanic and non-melanic females and males (Table 3). As for wing size, melanic females and males showed higher FA than non-melanic individuals (Fig. 5b).

**Table 3:** Partitioning of directional (MS side) and non-directional (MS side × individual) (× 10^3^ for shape) asymmetry for melanic and non-melanic females and males of *T. infestans* collected in Cruz del Eje, Cordoba Province, Argentina, using two-way mixed and Procrustes ANOVAs

Group	Sex	Size			Shape		
		MS side	MS side × individual	Measurement error	MS side	MS side × individual	Measurement error
Non-melanic	Female	0.052	0.147***	0.000	0.486**	0.133***	0.000
	Male	0.968	0.053***	0.000	0.728**	0.105***	0.000
Melanic	Female	3.120**	2.604***	0.006	0.634	0.517***	0.000
	Male	3.367**	0.194***	0.000	1.089*	0.150***	0.000

*P < 0.05, **P < 0.01, ***P < 0.001

## Discussion

Our results showed the existence of a measurable colorimetric variation among the sampled adults of *T. infestans* collected mainly in peridomestic habitats. To our knowledge, this is the first time that color variation is quantified accurately in a species of the Triatominae. The technique allowed us to group the individuals into two color morphs (i.e. melanic and non-melanic groups) and emerges as a promising tool to study this kind of variation in triatomines, as previously shown in other insect species [38]. Fine-scale quantification of phenotypic color variation has potential applications in the study of populations, sibling species, hybrids and closely related taxa [1, 3, 4].

This is the second report of melanic forms of *T. infestans* in domestic and peridomestic habitats in the Dry Chaco region of Argentina. Our results showed that melanic adults did not have an aggregated distribution, but were scattered throughout the study area; moreover, in 7 of the 13 infested houses where they were detected, they were collected along with non-melanic individuals. Taking into account that the last community-wide insecticide spraying campaign conducted before the study was three years earlier, house infestation and colonization (53% and 47%, respectively) were relatively high, indicating a good reestablishment of *T. infestans* colonies. This inference is based on the comparison with other endemic areas of the Argentine Chaco. For example, house infestation was 37% in a rural area located in the southern region of Los Llanos (La Rioja Province, Argentina) after decades of non-systematic vector surveillance and control [48]. In the locality of Pampa del Indio (Chaco Province, Argentina) house infestation was 31.9%, a much lower value than that expected from lack of recent insecticide spraying [49]. In an area in the Monte Desert ecoregion of mid-western Argentina, where the last insecticide spraying campaign conducted before the study was between 2 and 10 years earlier, depending on house accessibility, house infestation was 21.2% [50].

Heritability (h2) of melanization has been estimated in a few insect species (h2 = 0.73 in *Philaenus spumarius* [51], 0.77 in *Drosophila immigrans* [52], 0.17–0.37 in *Spodoptera littoralis* [53, 54] and 0.61 in *Gryllus firmus* [55]), indicating that this trait can respond rapidly to selection. In many species, melanism is genetically determined by only one or a few loci (e.g. Lepidoptera [56, 57], Diptera [58], Coleoptera [59, 60], Orthoptera [61, 62]). Moreover, it has been observed that the pattern of inheritance of melanism follows Mendel’s simple laws of segregation and an independent assortment of alleles in some species [63, 64], like the triatomine species *Rhodnius nasutus* [65]. In the latter case, the melanic phenotype also seems to be encoded by recessive alleles. The fact that in our study area melanic *T. infestans* adults were widely spread, with relatively high representation in the area at the house infestation level (45.83%) compared with non-melanic adults (83.33%), suggests that this trait may have been present in these populations for a sufficient number of generations to be well established and represented in this area. Chromatic variants have been reported in other Triatominae species. In *Triatoma rubrovaria*, four discrete chromatic morphotypes were identified in the same geographical area in the State of Rio Grande do Sul, Brazil [66]. In this case, chromatic variation was represented by patterns of discrete chromatic variants of the pronotum and the frequency of each morphotype was variable according to different ecotopes or municipalities in the same area. Chromatic phenotypes were studied in *Triatoma brasiliensis macromelasoma* in various locations between the distributions of *T. b. brasiliensis* and *T. juazeirensis.* Thirteen different color patterns were characterized, with nine of them being intermediate phenotypes [67]. For *Rhodnius nasutus*, the occasional appearance of adults with atypical dark coloring was observed in the F3 generation of a colony from Sobral, State of Ceará, Brazil. In this case, dark and typical morphs were discrete color variants easily identifiable in both nymphs and adults [65].

Environmental factors are considered determinant of variation within structures or traits. Insects generally grow to smaller sizes at high temperatures [68] and on low quality diets [69]. However, how these environmental signals alter the developmental programme to produce smaller individuals is still unknown. Our results showed that melanic females and males exhibited larger body size, body area and wings than the non-melanic ones (although this tendency was not significant for body size or body area in females). In the Triatominae, evidence of size reduction was reported for wings of domestic specimens of *T. infestans* collected from two sites in Cochabamba, Bolivia (Laguna Angostura and Jamach’uma) compared with sylvatic dark morphs collected 1 km away from these domestic foci [70]. A reduction in body size was also observed in *Panstrongylus rufotuberculatus* (Triatominae) in the transition from sylvatic to domestic habitats [71] and from natural to artificial (laboratory) habitats for *Panstrongylus geniculatus*, *Triatoma brasiliensis* and *Triatoma flavida* [72, 73]. Considering that in our study area there is no geographical or even physical separation (in seven houses melanic and non-melanic individuals were collected in the same sites) between collected melanic and non-melanic individuals, differences in wing size may be attributed to differences in ecological adaptation of both colorimetric groups to the same habitats. It has been postulated that melanism is an important trait related to thermoregulation, with darker individuals probably heating up faster and reaching higher body temperatures than light-colored ones due to the higher absorption of solar radiation [74]. Thermal capacity is strongly related to body size; larger bodied individuals would exhibit lower cooling rates (higher heat conservation) but also lower heating rates (lower capacity to gain heat) than smaller bodied ones.

Shape variation in *T. infestans* is considered frequently driven by genetic variations [75]. Wing shape differences between groups showed that these populations are structured, suggesting different origins for both morphs. The fact that the area of study had been treated with insecticide three years before this study suggests that *T. infestans* from both colorimetric groups may have become from different sources (non-treated nearby areas and/or residual or untreated foci [76–79]). A strong spatial structuring at a microgeographical scale using either phenotypic or genotypic markers after community-wide or selective residual spraying with insecticides has been reported for *T. infestans* populations in other areas of the Dry Chaco [80–82]. Considering that starved individuals would disperse to locate eventual blood sources, high population structuring might be expected to be associated with high nutritional status [83]. Nutritional status is, among others, a factor that modulates flight dispersal in the Triatominae [31]. For *T. infestans*, many authors use the W/L ratio as an indicator of flight initiation [30–33]. Our results showed that this ratio did not differ between groups. For *T. infestans*, adults with W/L < 8 mg/mm have high probability of flight initiation [30, 31]. The W/L ratio for the adults analyzed in this study varied between 7.10–17.41 mg/mm. Eight (8.7%) of the adults exhibited a W/L ratio < 8 mg/mm, of which 6 (75%) were non-melanic and 7 (87.5%) were males. Despite these observations, within each house, results showed no significant differences in W/L between melanic and non-melanic groups, confirming that flight initiation is not associated with a particular colorimetric group.

Our results showed that melanic females and males exhibited significantly higher FA indices than non-melanic ones (24.6% and 69.9% higher for wing size and shape for females, respectively, and 72.7% and 34.8% higher for wing size and shape for males, respectively). This apparent difference in the magnitude of FA between colorimetric groups was also observed in *T. infestans* collected in different ecotopes, seasons and years, and before and after a community-wide pyrethroid spraying campaign in rural areas of Argentina [84, 85]. In these studies, different developmental instability suggests congruence of FA with habitat/host combinations and/or timing of flight dispersal for the former example [84] and differential survival of adults with more symmetric wings for the latter study [85]. In the context of the present study, differences in wing developmental instability suggest a different influence of environmental conditions during development between morphs. It is well known that there is a wide range of phenotypic effects correlated with melanism, including developmental time, behavioural traits and different tolerance to environmental characteristics, such as temperature, UV radiation and relative humidity (see Table 2c in [1]). For example, in *Manduca sexta*, melanic forms are less tolerant to desiccation than typical non-melanic individuals [5]. Environmental characteristics of the different domestic and peridomestic habitats where individuals develop might have a differential effect on the level of instability of melanic and non-melanic individuals. Another possibility that may explain the differences found in FA indices could be associated with the general hypothesis that melanization is costly and will result in trade-offs [1, 54]. This hypothesis is based on two premises: first, tyrosine, the main compound of the pro-phenol oxidase system, can only be obtained from ingested food; secondly, melanin is a nitrogen-rich compound that may require substantial nitrogen or protein investment for its synthesis [86]. Several traits described in the literature as associated with melanism are likely to have negative consequences on fitness [1]. In this sense, melanic individuals should be less adapted to domestic and peridomestic habitats where non-melanic ones seem to be better adapted, considering the recorded levels of FA. While the physical attributes of melanism may have obvious selective advantages (e.g. thermal regulation), the effect of energetic costs on fitness-related traits such as fecundity depends on how such costs interact with these traits. Moreover, dispersive capacity of melanic individuals may be mechanically compromised due to their less symmetrical wings, considering flight performance and wing function [87].

Our results showed that although chromatic cluster analysis clearly defined a melanic and a non-melanic group, within the non-melanic group, there are also two well-differentiated groups by the PC2 (cluster 2 and 3, Additional file 1: Figure S1). These two groups showed significant differences in total body length and total body area for females and significant differences in wing shape for males. Fluctuating asymmetry analysis showed differences between groups (4.6% and 2.4% for wing size and shape for females, respectively, and 3.2% and 5.8% for wing size and shape for males, respectively). These results indicate that differences between groups were not always consistent between sexes and do not allow us to draw a clear conclusion about whether the color variants within the non-melanic groups can be attributed to different origins of reinfestation or are only a common colorimetric variants exhibited by a population.

To the best of our knowledge, this study is a first approach to the melanic and non-melanic characterization in *T. infestans* morphs associated with domestic and peridomestic structures. Understanding other pleiotropic effects associated with melanism, identification of responsible genes of melanism and selective responses of melanic alleles in nature, among other factors, would help elucidate the evolutionary history, the epidemiological importance and the possible origins of melanic forms in domestic and peridomestic *T. infestans* populations.

## Conclusions

Two colorimetric groups of *T. infestans* were recorded in a rural area from Cruz del Eje, Córdoba Province, Argentina, with a high infestation rate (53%). Melanic adults were widespread throughout the study area, although less represented than non-melanic ones. Linear and geometric morphometric analyses showed a greater body size for melanic females and males than for non-melanic ones, suggesting possible differences in ecological adaptation. Wing shape analysis revealed differences between groups, which may be related to their different origins. Melanic and non-melanic individuals exhibited good nutritional status and low probability of flight initiation (W/L > 8 mg/mm). Wing size and wing shape FA results are in agreement with lower adaptation of melanic individuals to domestic and peridomestic environments or a cost associated with melanism. This study provides evidences of a well-established *T. infestans* chromatic polymorphism in the area and possible pleiotropic effects of melanism associated with environmental conditions.

## Supplementary information

**Additional file 1: Figure S1.** K-means clustering analysis. **a** Plot of the curve of the average silhouette for 10 K-values (silhouette method). The optimal number of clusters suggested by this method (three) is indicated with a dashed line. **b** Biplot derived from the first two axes of a principal component analysis showing the spatial distribution of the three clusters derived from a K-means clustering analysis (K = 3).

## Data Availability

The datasets supporting the conclusions of this article are included within the article and its additional file. Raw data are available from the corresponding author upon reasonable request.

## Abbreviations

APDI: adult peridomestic infestation
CS: centroide size
DA: directional asymmetry
FA: fluctuating asymmetry
h2: heritability
IDI: infestation in the intradomicile
IDC: colonization in the intradomicile
L: total body length
ME: measurement error
PDI: infestation in the peridomicile
PDC: colonization in the intradomicile
RGB: red, green, blue
W: weight
W/L: total weight/total body length ratio
